# Reliability and Validity of the Chinese Version Appropriateness Evaluation Protocol

**DOI:** 10.1371/journal.pone.0136498

**Published:** 2015-08-25

**Authors:** Wenwei Liu, Suwei Yuan, Fengqing Wei, Jing Yang, Zhe Zhang, Changbin Zhu, Jin Ma

**Affiliations:** 1 Antai College of Economics and Management, Shanghai Jiao Tong University, Shanghai, China; 2 School of Public Health, Shanghai Jiao Tong University, Shanghai, China; 3 Department of Medical Administration, Shanghai Rui Jin Hospital, Shanghai, China; 4 Department of Pathology, Erasmus Medical Center, Rotterdam, The Netherlands; University of Chieti, ITALY

## Abstract

**Objective:**

To adapt the Appropriateness Evaluation Protocol (AEP) to the specific settings of health care in China and to validate the Chinese version AEP (C-AEP).

**Methods:**

Forward and backward translations were carried out to the original criteria. Twenty experts participated in the consultancy to form a preliminary version of the C-AEP. To ensure applicability, tests of reliability and validity were performed on 350 admissions and 3,226 hospital days of acute myocardial infraction patients and total hip replacement patients in two tertiary hospitals by two C-AEP reviewers and two physician reviewers. Overall agreement, specific agreement, and Cohen’s Kappa were calculated to compare the concordance of decisions between pairs of reviewers to test inter-rater reliability and convergent validity. The use of “overrides” and opinions of experts were recorded as measurements of content validity. Face validity was tested through collecting perspectives of nonprofessionals. Sensitivity, specificity, and predictive values were also reported.

**Results:**

There are 14 admission and 24 days of care criteria in the initial version of C-AEP. Kappa coefficients indicate substantial agreement between reviewers: with regard to inter-rater reliability, Kappa (κ) coefficients are 0.746 (95% confidence interval [CI] 0.644–0.834) and 0.743 (95% CI 0.698–0.767) of admission and hospital days, respectively; for convergent validity, the κ statistics are 0.678 (95% CI 0.567–0.778) and 0.691 (95% CI 0.644–0.717), respectively. Overrides account for less than 2% of all judgments. Content validity and face validity were confirmed by experts and nonprofessionals, respectively. According to the C-AEP reviewers, 18.3% of admissions and 28.5% of inpatient days were deemed inappropriate.

**Conclusions:**

The C-AEP is a reliable and valid screening tool in China’s tertiary hospitals. The prevalence of inappropriateness is substantial in our research. To reduce inappropriate utilization, further investigation is needed to elucidate the reasons and risk factors for this inappropriateness.

## Introduction

Appropriateness of utilization in healthcare requires accessibility, cost-efficiency, and quality [1, 2]. For the long-term, the issue of focus and discussion in public policy is mainly the absence of accessibility. China, like many other developing countries, tends to emphasize universal access to sufficient health resources. From 2008 to 2012, the coverage of health insurance rose from 87.9% to over 95%. In the meantime, however, healthcare expenditures almost doubled. In 2012, health costs represented about 5.4% of GDP in China. The annual growth rate of total healthcare expenditure was around 15% in the last decade [3, 4]. Although the amount is not considerable compared to that in developed countries, the rapid growth is worth noting. Containing escalating healthcare costs has become a major issue for policy-makers. In China, hospitals play the dominant role in healthcare delivery. It is reported that over 60% of total healthcare expenditure was spent in hospitals, and 71.3% of inpatients were admitted in hospitals in 2012. From 2008 to 2012, the average growth of public hospital expenditure was 19.6%, which exceeded that of total healthcare costs [3]. With the rapid increase of hospital use, irrational utilizations, such as prolonged length of stay and admission of non-acute conditions, are widely observed [5]. From 2008 to 2011, outpatient visits only increased by 0.7%. Meanwhile, the number of hospital admissions increased by 8.5% annually [6]. With regard to hospital stay, according to the China Health Statistical Yearbook, the average length of stay (ALOS) in hospitals was 10.0 days in 2012, while OECD countries are reported to have an ALOS of about eight days (2011) for acute care [3, 7]. All of the above figures underline the pivotal importance to rationalize hospital use in China.

To contain healthcare costs generated by inappropriate use, utilization review (UR) programs have been designed and conducted in many countries. UR targets to reduce medically unnecessary care by determining whether a service is carried out according to the appropriateness of intensity and cost [8]. The practices of UR were initiated in the U.S. in the 1980s, and this effort was followed by many countries [9]. The essential aspect of UR is the use of a reliable and valid rating judgment system to make decisions on the appropriateness of the review unit. Among all of the rating tools, the Appropriateness Evaluation Protocol (AEP) is one of the most widely adopted, and has been assessed to have relatively satisfactory metric properties [10]. The original AEP comprises two major parts, admission and days of stay. Each part contains a list of reasons to justify the appropriateness of a single admission or a hospital day by severity of patient condition and intensity of services. If any one of the criterion is met, the admission or the index day can be evaluated as appropriate. In addition to the objective list of criteria, the AEP also has a subjective part, called the “override” option. This option allows the reviewer to determine an admission or an inpatient day to be appropriate even if no criterion is met or to deny the appropriateness of the reviewed unit even if one or more items can be applied [9]. Considering the differences in healthcare systems, economies and cultures, the AEP was implemented with modifications in many countries and regions [11–19].

However, the prevalence of UR projects did not take place in China. In fact, only 11 domestic studies on the evaluation of hospital use were found through a thorough search of the relevant literature [20–30]. According to the results of eight studies which adopted the AEP as the screening tool, the rates of inappropriate admission range from 14.6% to 34.5%, and the percentage of inappropriate hospital days varies from 8.5% to 24.2% [23–30]. Although these studies revealed the existence of inappropriate use and the applicability of appropriateness review in China to some extent, it is worth noting that there are two major flaws of their study design. First, of the eight AEP studies, except for one study which was conducted in township hospitals [29], all of the others adopted the Chinese translation of the AEP directly without adaptation, and no test of reliability or validity was performed. Second, in six of the studies, the screening criteria were applied indiscriminately to pediatric and adult patients [21, 23–25, 29]. These deficiencies made the conclusions less valid.

Our study aims to accommodate the criteria of the original AEP to the specific context in China and to test its applicability in two tertiary hospitals in Shanghai.

## Methods

### Tool development

The first step of tool adaptation involves translating the original items into Chinese. A bilingual MD was recruited to perform the translation from English to Chinese, and then a physician conducted a backward translation for confirmation. Differences in translation were compared and discussed until a consensus was reached.

To adapt the criteria to China’s medical settings, seven experts from the department of internal cardiology, six from the department of cardiac surgery, and seven from the department of orthopedics were invited to participate in our study in July 2014. Each of the 20 experts was required to have over 10 years of clinical experience. In the first phase, questionnaires of all AEP screening items in Chinese were distributed to the panel. Following each item, there were options of “retain” and “delete”, and the respondents were asked to make their decisions using their own medical knowledge and experience independently. The “override” option was not included in the first survey to assure the comprehensiveness of the C-AEP. The agreement rate of each item was calculated. If an item receives 75% of agreement of “delete”, the decision of deletion will be suspended to the next stage. In the second round of consultancy, questionnaires of the retained items were handed out to the panel. They were then required to answer the questions regarding whether a specific criterion should be modified according to the specific settings of China and whether the final decision of deletion should be executed. Considering the difficulty of reaching a consensus, the same rule of 75% agreement was adopted. Open questions were included in both investigations.

To enhance the operability in review practice, a pilot study on a randomly sampled 10 records was conducted. If difficulty existed in the review process, the item being questioned by the C-AEP raters would be discussed with the expert panel for amendment.

### Study population

Two teaching hospitals agreed to join our research. The two hospitals are typical public tertiary hospitals with bed-size of over 1,800 and 2,300, respectively. Of all discharged records from the departments of cardiology, cardiac surgery, and orthopedics in 2013 (Hospital A) or 2014 (Hospital B), the records of patients with a provisional diagnosis of acute myocardial infarction (AMI) or receipts of total hip replacement (THR) were extracted from the electronic medical archives management system. The cases selected for review were included in the hospital quality management program and are of sufficient inpatient populations. Records of patients under 16 years old or who had less than 24 hours of stay were excluded. 1,059 eligible admissions (775 for AMI and 284 for THR) and 12,966 patient days (8,798 for AMI and 4,168 for THR) were listed. Two independent systematic samplings were carried out for admissions and hospital days. For admissions, we randomly selected two days per week through 2013 and 2014, and all records of the patients admitted on the indexed weekday/weekend were sampled. Similarly, all patient days on the indexed two days of each week were to be reviewed. For example, if the sampled days are Wednesday and Saturday in the first week for patient days, all records of the patients who stayed in the hospital on those two days would be drawn [31]. However, since the review unit is an admission or a single hospital day, only the admission day or patient day were examined, not the whole stay. In addition to the consideration that every weekday and weekend should be evenly distributed, the sample size was also decided according to the contracted workload for the reviewers.

### Reliability and validity

To test the inter-rater reliability, which refers to the concordance degree of assessments between raters who use the same instrument, 350 admissions and 3,226 patient days were reviewed by two primary investigators using the C-AEP independently in three months. The investigators were research assistants with previous experience in application of the original AEP [26]. A brief training on employment of the rating tool for one day was provided. The judgments of the assessors were compared by means of overall agreement, specific agreement, and Cohen’s Kappa for overall agreement.

Convergent validity measures to what extent the judgments made according to the instrument comply with those made by another method. In our study, this was assessed by comparing the agreement rates between the physician reviewers and the primary reviewers. Two physician reviewers were recruited to assess the same sample of the reliability study by working together. One of them was not working for any of the participating hospitals, and the other, who works at one of the hospitals as a surgeon, claimed to have no influence on the admission or treatment of the sampled patients. After an intense training in the appropriateness in healthcare and the use of the rating form for two days, the physician reviewers began to review the indexed units according to their own professional knowledge without the awareness of the C-AEP. After all of the ratings were completed, the research team asked the C-AEP raters to reach a consensus wherever disagreement existed in the inter-observer reliability study, and the comparison of agreement was calculated between the judgments of the physicians and the consensus of the two primary reviewers. This approach retained the hypothesis power by limited raters [32, 33], and it resembles the actual review process which requires two reviewers to reach a mutual agreement [34]. The comparison of judgments between raters is presented by means of overall agreement, specific agreement, and Kappa value. The use of the override option by the trained investigators and inquiries of the expert panel were recorded as measurements of content validity. Face validity was also evaluated by referring to nonprofessionals. Sensitivity, specificity, positive predictive value, and negative predictive value were computed in the same frame of validity assessment.

### Statistics

Overall agreement, specific agreement, and Cohen’s Kappa between different groups of raters were computed as measurements of inter-rater reliability and convergent validity. Overall agreement rate is the proportion of the same judgments made by reviewers of all ratings; whereas, specific agreement or effective percentage of agreement rate stands for the percentage of agreement when at least one reviewer determines that the unit is inappropriate. To factor out agreement by chance, Cohen’s κ is a suitable statistic that measures the true extent of overall agreement between raters.

Landis and Koch’s guidelines were adopted as benchmark scales of Cohen’s Kappa. A κ coefficient over 0.61 is considered to indicate a substantial agreement rate [35]. The use of the override option should not be over 5% to ensure acceptable validity [15, 36].

The Academic Ethics Board of each hospital approved this retrospective study. The two hospitals are teaching hospitals (affiliated with Shanghai Jiao Tong University) that provide both care services and health related education. Once a patient is admitted to the hospitals, the agreement of providing his/her personal and medical information for academic use is established by mutual consent. We also signed a contract with the Academic Ethics Board of each hospital to guarantee that the data shall be used only for academic purpose. In addition, the medical records were anonymized and de-identified through the review and analysis procedure.

## Results

### The C-AEP

Moderate modifications of the AEP were made by the consensus of the expert panel. In the first round expert consultation, five criteria were suspended through deletion from the protocol. These criteria are “intravenous medications and/or fluid replacement (does not include tube feedings)”, “intramuscular antibiotics at least every 8 hours”, “biopsy of internal organ that day”, “intramuscular and/or subcutaneous injections at least twice daily”, and “occurrence of a documented new AMI or cerebrovascular accident (stroke) within 14 days before the day of review”. The first two items are from the admission criteria battery, and the latter three pertain to the day of care section. The agreement rates of “delete” are 90%, 75%, 85%, 75%, and 90%, respectively (Table 1). The reasons for deletion presented by the experts are “lack of severity”, “can be done by an outpatient department”, and “shall not be applied alone”. No alterations were made in the second round, and the deletions pending from the previous survey were approved by consensus. In our preliminary version, the C-AEP is structured the same as the U.S. version, but it contains 14 admission criteria and 24 days of care criteria, instead of 16 and 27, respectively, in the original protocol [9] (S1 Text).

**Table 1:** Deleted items and agreement rates of retention.

Deleted criteria	Agreement rates (%)
Intravenous medications and/or fluid replacement (does not include tube feedings)	10
Intramuscular antibiotics at least every 8 hours	25
Biopsy of internal organ that day	15
Intramuscular and/or subcutaneous injections at least twice daily	25
Occurrence of a documented new AMI or cerebrovascular accident (stroke) within 14 days before the day of review	10

The expressions of six items (A6, B7, B9, B14, B16, and B20) were slightly altered after the pilot assessment to accommodate them to medical practices in China. For example, the temperatures in the original AEP were described in Fahrenheit, while Celsius is more common in the records of China’s hospitals (S1 Text). However, although adjustments were performed to facilitate interpretation for the reviewers, no fundamental changes were made.

### Reliability

Overall agreement, specific agreement, and Cohen’s Kappa were utilized to assess inter-observer reliability (Table 2). The overall agreements for all admissions and index days were 93.4% and 90.3%, respectively, and the specific agreements were 64.6% and 67.6%, respectively. The κ values for admission and inpatient days were 0.746 (95% Confidence Interval [CI] 0.644–0.834) and 0.743 (95% CI 0.698–0.767), respectively. Agreement statistics show that the agreements for THR were generally higher than those for AMI cases, and the overall agreements were higher than specific agreements. The overall agreements in admission for AMI and THR were 93.0% and 94.4%, respectively; the overall agreement levels for days of care were 89.0% and 92.5%, respectively. Specific agreements in admission for AMI and THR were 57.5% and 76.0%, respectively; specific agreements in hospital days were 58.7% and 78.4%, respectively. The κ values of THR units were also greater than those of AMI. The κ values of admission were 0.690 (95% CI 0.551–0.815) and 0.828 (95% CI 0.669–0.946) for AMI and THR, respectively; the κ values of days of stay were 0.672 (95% CI 0.579–0.711) and 0.825 (95% CI 0.760–0.858) for AMI and THR, respectively.

**Table 2:** Inter-rater agreements of C-AEP reviewers.

	AMI		THR		All	
	Admission (n = 243)	Days of stay (n = 1973)	Admission (n = 107)	Days of stay (n = 1253)	Admission (n = 350)	Days of stay (n = 3226)
Overall agreement (%)	93.0	89.0	94.4	92.5	93.4	90.3
Specific agreement (%)	57.5	58.7	76.0	78.4	64.6	67.6
κ(SE)	0.690 (0.070) ^1^	0.672 (0.020) ^1^	0.828 (0.068) ^1^	0.825 (0.017) ^1^	0.746 (0.050) ^1^	0.743 (0.014) ^1^
κ CI 95%	0.551–0.815	0.579–0.711	0.669–0.946	0.760–0.858	0.644–0.834	0.698–0.767

^1^ *p*<0.001

### Validity

The use of the override option accounted for 1.7% (n = 6) and 1.1% (n = 4) of admission judgments; for inpatient days, the percentages were 1.6% (n = 53) and 1.0% (n = 34) for each C-AEP reviewer. The reviewers both reported that the overrides were employed mainly because of the lack of documented information to process, and they had to refer to additional information (i.e., test records, surgical sheets, physician orders, etc.) for decision-making. Content validity was also assured by the expert panel during and after the tool development phase. No request for adding a new criterion to the list was recorded, and no difficulty was reported in interpretation of the descriptions.

Face validity was tested by a semi-structured survey with the translators. They both provided positive responses in regard to their perceived function and usefulness of the tool.

Convergent validity was assessed by the agreement rates between the judgments of physician reviewers and the consensuses of the primary reviewers. The overall agreements for all reviewed admissions and hospital days were 90.9% and 87.0%, respectively, and the specific agreements were 57.9% and 64.4%, respectively. The Kappa statistics, 0.678 (95% CI 0.567–0.778) for admissions and 0.691 (95% CI 0.644–0.717) for patient days, were slightly lower than those for the reliability study. The overall agreements of admission for AMI and THR were 89.7% and 93.5%, respectively, and the κ values were 0.614 (95% CI 0.463–0.750) and 0.797(95% CI 0.632–0.923), respectively. Specific agreements of admission were 51.0% and 72.0%, respectively. For days of stay, the overall agreement rates were 84.4% and 91.0% of AMI and THR, respectively; the κ values of overall agreement were 0.617 (95% CI 0.531–0.652) and 0.796 (95% CI 0.732–0.832), respectively. The specific agreements were 56.7% and 76.0% for each patient group, respectively (Table 3).

**Table 3:** Inter-rater agreement of physician raters and C-AEP reviewers.

	AMI		THR		All	
	Admission (n = 243)	Days of stay (n = 1973)	Admission (n = 107)	Days of stay (n = 1253)	Admission (n = 350)	Days of stay (n = 3226)
Overall agreement (%)	89.7	84.4	93.5	91.0	90.9	87.0
Specific agreement (%)	51.0	56.7	72.0	76.0	57.9	64.4
κ (SE)	0.614 (0.070)^1^	0.617 (0.020) ^1^	0.797 (0.073) ^1^	0.796 (0.018) ^1^	0.678 (0.053) ^1^	0.691 (0.014) ^1^
κ CI 95%	0.463–0.750	0.531–0.652	0.632–0.923	0.732–0.832	0.567–0.778	0.644–0.717

^1^ *p*<0.001

The results also signify that the C-AEP is very sensitive and relatively specific, with satisfactory predictive power. For admissions, the sensitivity and specificity were measured to be 93.2% and 78.6%, respectively. Regarding patient days, the measurements were calculated to be 92.7% and 74.6% for each review object, respectively. Positive predictive and negative predictive values were 95.8% and 68.8% for admission, respectively, and were 88.8% and 82.5% for hospital days, respectively.

### Inappropriate utilization levels

According to the consensus of the C-AEP reviewers, of the 350 admissions and 3,226 days of care, 18.3% of the admissions and 28.5% of the inpatient days were inappropriate. The results are tabulated in Table 4. 16.5% and 25.6% of the admissions were categorized as inappropriate use for AMI and THR, respectively. Compared to the inappropriateness rate of 25.6% for AMI, the inappropriate days accounted for a larger scope of 33.1% for THR (Table 4).

**Table 4:** Inappropriate rates by C-AEP reviewers.

	AMI		THR		All
	n	%	n	%	%
Admission	40	16.5	24	22.4	18.3
Days of stay	506	25.6	415	33.1	28.5

## Discussion

Only small modifications of the original AEP were made in the tool development stage. The original protocol was designed under the U.S. settings in the 1980s [9]. Previous experience from other countries shows that it is not imperative to make major changes to the protocol to ensure applicability, and the adaptions made by these studies were primarily small scales of deletion, item adding, and wording [14–17, 37–39]. The adjustments of items can be justified by differences in healthcare delivery systems and improved medical technology. In our study, the deletions can also be attributed to the scarcity of high-quality healthcare resources. It is observed that the issue of the difficulty of obtaining access to inpatient care persists in China, and the phenomenon of shortages in the numbers of beds is common in almost all tertiary hospitals [40].

The C-AEP is a reliable and valid appropriateness rating instrument for China’s hospitals. The usefulness of the AEP is frequently questioned [41, 42]. In order to validate the C-AEP, forward and backward translation, expert panel discussion, intensive training session for reviewers, pilot evaluation, and agreement tests were performed sequentially. According to the results of our study, the magnitudes of overall agreement are considerable, and the Kappa values for admission and patient days are both substantial according to the adopted benchmarks. Kappa statistics were found to be almost perfect in both admission and patient days of THR in the reliability study. However, the specific agreement for admission is relatively lower. This may due to the acute nature of the selected case-mix and procedure, and the low prevalence of negative judgments has an impact on the degree of calculated agreement. The analysis of sensitivity, specificity, and predictive capability also suggests that the C-AEP is of high referral value in screening inappropriate utilization.

It is conceivable that the level of inappropriateness found in days of stay by the C-AEP is higher than those in other studies since the length of stay is generally longer. Although comparing the outcomes of our study with those in other settings (mainly developed countries) may lack a rational basis, the level of inappropriateness calls for further exploration. Our preliminary assumption is that the imbalanced reimbursement policy, inconsistent healthcare delivery system, and cultural differences are the main causes. First, expanding coverage of health insurance promotes utilization of healthcare resources [43]. Since no adequate control mechanism exists, overuse of care can be easily encouraged by providers under the fee-for-service payment method [4]. Moreover, the general situation of reimbursement in China is that out-of-pocket expenses are lower for insured inpatients than for outpatients. In fact, some services are not even covered in the outpatient scheme [43]. Second, the inconsistency of China’s healthcare delivery system undermines the efficiency of care delivery. Without an integral referral system, post-procedure recovery can only be done in a hospital [4]. For example, in our study, the length of stay of THR is 14.7 of the sampled hospitals, while the ALOS is reported to be 10.5 in Spain (2008) and 4.2 in the U.S. (2002–2007) [44, 45]. For AMI patients, the ALOS is 11.3 in our sample frame, while the number for OECD countries is around seven [7]. In addition, the fact that nearly one-third of the hospital days were unjustified according to the C-AEP can also provide support for this assertion. Third, it should be noted that cultural factors may also play a role in the variance of ALOS. It is observed that inpatients in Asian countries (e.g., Japan and Korea) have a relatively longer stay than in other OECD countries [7]. In our initial trial of tool development and validation, only clinical criteria of the original AEP were included, and it is premature to draw any final conclusions. In our larger investigation, when an admission or a day of care failed to meet any requirement of the items, the reviewer referred to a list of reasons for the inappropriate use. The list of reasons, which was considerably modified in the European version, was not presented as a part of this research [46]. A follow-up study will be performed in order to complete the effort to reduce inappropriate hospital use in China.

The results of retrospective appropriateness auditing by the C-AEP rely heavily on the quality of patient records. The two hospitals in this study are large-scale general hospitals, both of which have been involved in a quality management program for selected case-mixes and procedures since 2010. The project requires the participants to collect data regarding care delivery processes and outcomes for quality assurance. Thus, the electronic patient records in our study are well structured, and all paper charts were scanned clearly. Although evidence supports our conclusion that the C-AEP is reliable and valid in specific settings, it is possible that the variance in the quality of documents in some other hospitals would have a negative influence on the review process [15]. Further evidence is needed to demonstrate its usefulness in other contexts (hospitals located elsewhere or other departments). Pilot research is strongly recommended for all projects that use the C-AEP, and formatted reporting data as proposed by Ash should be considered [31].

## Conclusion

The C-AEP is a reliable and valid screening tool in China’s tertiary hospitals. Although few revisions were made to the original version, the results reveal the necessity of adaptation before application. The preliminary results show a substantial proportion of inappropriate utilization in hospitals. To find effective countermeasures for reducing inappropriate use, further investigation is needed to elucidate the reasons and risk factors for unnecessary care.

## Supporting Information

S1 Text C-AEP Criteria. (DOCX)

S2 Text Web Links to Chinese References. (DOCX)
