# Supplementary material for: Reliability and Validity of the Chinese Version Appropriateness Evaluation Protocol
Source: PLoS One. 2015 Aug 25;10(8):e0136498. doi: 10.1371/journal.pone.0136498 (PMC4549286; doi:10.1371/journal.pone.0136498)
Supplement: S1 Text — (DOCX) [file pone.0136498.s001.docx]

# C-AEP Criteria

**Admission criteria**

**Severity of illness**

A1. Sudden onset of unconsciousness or disorientation (coma or unresponsiveness)

A2. Pulse rate: a. <50 per minute or b. >140 per minute

A3. Blood pressure:

a. Systolic <90 or >200 mmHg

b. Diastolic <60 or >120 mmHg

A4. Acute loss of sight or hearing

A5. Acute loss of ability to move body part

A6. Persistent fever >37.8℃ (by mouth) or >38.3℃ (rectally) for more than 5 days

A7. Active bleeding

A8. Severe electrolyte/blood gas abnormality (any of the following):

a. Na < 123 mEq/L; Na > 156 mEq/L

b. K < 2.5 mEq/L; K > 6.0 mEq/L

c. CO_2_ combining power (unless chronically abnormal) < 20 mEq/L; CO_2_ combining power (unless chronically abnormal) > 36 mEq/L

d. Arterial pH < 7.30 Arterial pH > 7.45

A9. EKG evidence of acute ischemia; must be suspicion of a new myocardial infarction

A10. Wound dehiscence or evisceration

**Clinical Services**

A11. Surgery or procedure scheduled that day requiring:

a. General or regional anesthesia and/or b. Equipment or facilities available only for inpatients

A12. Vital sign monitoring every 2 hours or more often (may include telemetry or bedside cardiac monitor)

A13. Chemotherapeutic agents that require continuous observation for life-threatening toxic reaction

A14. Intermittent or continuous respirator use at least every 8 hours

**Days of stay criteria**

**Medical Services**

B1. Procedure in operating room that day

B2. Scheduled for procedure in operating room the next day, requiring preoperative consultation or evaluation

B3. Cardiac catheterization that day

B4. Angiography that day

B5. Thoracentesis or paracentesis that day

B6. Invasive central nervous system diagnostic procedure (e.g., lumbar puncture, cysternal tap, ventricular tap, Pneumoencephalography) that day

B7. Any test or treatment that requiring strict dietary control, for the duration of the diet

B8. New or experimental treatment requiring frequent dose adjustments under direct medical supervision

B9. Close medical monitoring by a doctor at least three times daily

B10. Postoperative day for any procedure covered in number 1 or 3-6 above

**Nursing/Life Support Services**

B11. Respiratory care-intermittent or continuous respirator use and/or inhalation therapy (with chest PT, IPPB) at least thrice daily

B12. Parenteral therapy-intermittent or continuous IV fluid with any supplementation (electrolytes, protein, medications)

B13. Continuous vital sign monitoring-at least every 30 minutes, for at least 4 hours

B14. Intake and output measurement under doctor's orders

B15. Major surgical wound and drainage care (chest tubes, T-tubes, hemovacs, Penrose drains)

B16. Close medical monitoring by nurse at least three times daily, under doctor's orders (except routine checks of temperature and blood pressure)

**Patient Condition Factors**

(Within 24 hours before day of review)

B17. Inability to void or move bowels (past 24 hours) not attributable to neurologic disorder

(Within 48 hours before day of review)

B18. Transfusion due to blood loss

B19. Ventricular fibrillation or ECG evidence of acute ischemia, as stated in progress note or in ECG report

B20. Fever at least 38.3℃ rectally (at least 37.8℃ orally), if patient was admitted for reasons other than fever^1^

B21. Coma-unresponsiveness for at least one hour

B22. Acute confusional state, not due to alcohol withdrawal

B23. Acute hematologic disorders, significant neutropenia, anemia, thrombocytopenia, leukocytosis, erythrocytosis, or thrombocytosis yielding signs or symptoms

B24. Progressive acute neurologic difficulties
