# Supplementary material for: Reliability and Validity of the Chinese Version Appropriateness Evaluation Protocol
Source: PLoS One. 2015 Aug 25;10(8):e0136498. doi: 10.1371/journal.pone.0136498 (PMC4549286; doi:10.1371/journal.pone.0136498)
Supplement: S2 Text — (DOCX) [file pone.0136498.s002.docx]

# Web Links to the Chinese References

21. Wang X, Guo XE, Xu YY, Pan F, Liu L. [Ineffective hospitalization days and their influencing factors]. Journal of the Fourth Military Medical University. 2001;22(6):557-9. Chinese

<http://d.wanfangdata.com.cn/periodical_dsjydxxb_4200106023.aspx>

22. Wang H. [Influence of valueless period of hospitalization to period of hospitalization and costs]. Chinese Hospital Management. 2002;22(5):17-9. Chinese.

<http://d.wanfangdata.com.cn/periodical_zgyygl200205008.aspx>

23 Liu X, He MQ. [Study on hospitalization days appropriateness evaluation based on AEP]. Chinese Hospitals. 2008;12(10):31-4. Chinese.

<http://d.wanfangdata.com.cn/periodical_zhonggyy200810009.aspx>

24. Feng H, Cao JW. [Study of unnecessary length of stay of ANFH patients]. Chinese Hospital Management. 2009;29(4):37-40. Chinese.

<http://d.wanfangdata.com.cn/periodical_zgyygl200904016.aspx>

25. Qiu YZ, Meng K, Li N. [Study of inappropriate LOS of patients with alcoholic liver cirrhosis]. Chinese Hospital Management. 2013;33(4):19-21. Chinese.

<http://www.cnki.com.cn/Article/CJFDTotal-YYGL201304011.htm>

26. Tao JJ, Luo ZC, Ma J. [Existence of unnecessary hospitalization in inpatient service provision]. Journal of Shanghai Jiaotong University (Medical Science). 2013;33(8):1146-9. Chinese.

<http://xuebao.shsmu.edu.cn/CN/article/downloadArticleFile.do?attachType=PDF&id=10065>

27. Zhou XY, Wang H, Zhu ZM, Chen RX. [The appropriateness of hospital admission for patients with COPD]. Chinese Health Quality Management. 2013;20(4):33-5. Chinese.

<http://www.cnki.com.cn/Article/CJFDTotal-WSJG201304016.htm>

28. Liu K, Zhang C, Jiang L, Kang JF. [Inappropriate hospitalization research of clinical priority diseases]. Chinese Hospital Management. 2014;34(1):58-60. Chinese.

<http://d.wanfangdata.com.cn/periodical_zgyygl201401022.aspx>

30. Zhang WT, Wang L, Han YL, Chen FX, Li J, Zhao H, et al. [Evaluation of the hospitalization appropriateness of a third-level hospital in 5 years based on AEP]. Chinese Hospital Management. 2014(3):38-40. Chinese.

<http://d.wanfangdata.com.cn/periodical_zgyygl201403013.aspx>
